# Adverse Childhood Experiences Predict Treatment Drop Out in Adolescents and Young Adults With Eating Disorders

**DOI:** 10.1002/eat.24558

**Published:** 2025-09-24

**Authors:** David Lindenbach, Amelia Austin, Manya Singh, Abigail Trebilcock, Asmita Bhattarai, Gisele Marcoux, Gina Dimitropoulos

**Affiliations:** ^1^ Mathison Centre for Mental Health Research and Education, Hotchkiss Brain Institute, University of Calgary Calgary Alberta Canada; ^2^ Pathways to Prevention: A Centre for Childhood Trauma, Hull Services Calgary Alberta Canada; ^3^ Department of Psychiatry Cumming School of Medicine, University of Calgary Calgary Alberta Canada; ^4^ Alberta Children's Hospital Research Institute, University of Calgary Calgary Alberta Canada; ^5^ Department of Community Health Sciences Cumming School of Medicine, University of Calgary Calgary Alberta Canada; ^6^ Calgary Eating Disorders Program Calgary Alberta Canada; ^7^ Faculty of Social Work, University of Calgary Calgary Alberta Canada

**Keywords:** abuse, adolescent, adverse childhood experiences, child maltreatment, eating disorders, neglect, trauma, treatment completion, treatment retention, young adult

## Abstract

**Objective:**

Childhood adversity is associated with elevated risk of developing an eating disorder. The objective of the current study was to examine whether different types of childhood adversity, such as parental separation or abuse exposure, predicted treatment completion in a sample of adolescents and young adults with eating disorders.

**Method:**

A retrospective chart review was conducted at an eating disorders clinic in Calgary, Canada. Childhood adversity was measured using the adverse childhood experiences (ACEs) scale. Eating disorders diagnoses were determined by physicians following a comprehensive assessment. Logistic regressions were performed with ACEs as predictors of likelihood to complete treatment and including diagnoses and age as covariates.

**Results:**

Data were analyzed for 128 patients aged 11–24. Higher ACE scores were associated with a reduced likelihood of completing treatment before and after adjusting for diagnosis and age (adjusted odds ratio = 0.76). Examining individual ACE items, people were less likely to complete treatment if they were exposed to verbal abuse, physical abuse, emotional neglect, or if they witnessed intimate partner violence or substance abuse in the home. However, after adjusting for diagnosis, age, and other ACE exposures, no individual ACE was an independent predictor of treatment completion.

**Discussion:**

This study suggests that ACE exposure increases youth attrition from eating disorders treatment in a stepwise manner. Treatment programs for youth with eating disorders should focus additional effort on early intervention for youth who have dealt with childhood adversities, potentially by implementing trauma‐informed practice into assessments and treatment.

**Summary**

- Patients in an eating disorders clinic were less likely to complete treatment if they experienced a higher number of adverse childhood experiences
- The impact of adverse childhood experiences may be cumulative since no individual exposure was an independent predictor of treatment completion after accounting for age, diagnosis, and other adversity exposures.

## 1 Introduction

The development of eating disorders (EDs) is influenced by a combination of developmental, genetic, psychological, and sociocultural factors (Treasure et al. 2020). Exposure to trauma and childhood adversity has been identified as a significant risk factor for EDs (Burdo et al. 2023; Rienecke et al. 2022). Longitudinal and cross‐sectional studies suggest that childhood traumatic events exert a cumulative impact, with each additional exposure causing additional impairments related to health, mental health, financial stability, educational achievement, and social support (Copeland et al. 2018; Senaratne et al. 2024). For patients with EDs, exposure to child maltreatment is associated with an earlier age of symptom onset, greater ED psychopathology and illness severity, and increased prevalence of psychiatric comorbidities (Molendijk et al. 2017). The prevalence of child maltreatment among those with EDs is higher than the prevalence in the general population and also exceeds the prevalence in those with non‐ED psychiatric illness (Molendijk et al. 2017; Pignatelli et al. 2017; Rienecke et al. 2022).

While there is a clear association between childhood adversity and the development of EDs, it is less established whether childhood adversity predicts likelihood to complete treatment. This is a critical topic given the high rates of drop out in ED treatment, which are estimated at 20%–40% (Linardon et al. 2018; Dejong et al. 2012). Two recent systematic reviews suggested that exposure to trauma is associated with greater ED treatment drop out (Convertino and Mendoza 2023; Day et al. 2024). However, across the seven empirical studies related to trauma exposure within these reviews, two limited their scope to childhood sexual abuse (Carter et al. 2006; Castellini et al. 2018), three examined a single ED diagnosis (Anorexia Nervosa in Carter et al. 2006; Bulimia Nervosa in Mahon, Bradley, et al. 2001; Mahon, Winston, et al. 2001), and six only included patients over the age of 16 (Calugi et al. 2018; Carter et al. 2006; Castellini et al. 2018; Mahon, Bradley, et al. 2001; Mahon, Winston, et al. 2001; Pingani et al. 2012).

This study tested the hypothesis that exposure to different types of childhood adversity would predict treatment completion in a transdiagnostic sample of adolescents and young adults with EDs. A better understanding of factors that contribute to treatment retention will support treatment planning, which can improve treatment outcomes.

## 2 Methods

### 2.1 Participants

The current study included retrospective data collected as part of routine clinical care at the Calgary Eating Disorder Program in Calgary, Canada. The program supports individuals 7 years of age and older who meet the *Diagnostic and Statistical Manual for Mental Disorders—5th Edition* (DSM‐5; American Psychiatric Association 2013) criteria for an ED. The Calgary Eating Disorder Program offers inpatient, outpatient, and day treatment services, including medical, psychological, psychiatric, and nutritional support in addition to group and family therapy. Treatment is individualized for each patient, based on symptom acuity and individual/family capacity.

Inclusion criteria for our sample were: (1) individuals aged 7–24 years upon entry into the program, (2) DSM‐5 diagnosis of an ED, (3) completion of the adverse childhood experiences (ACEs; Felitti et al. 1998) scale with individual and total scores available, and (4) file closed between February 2016 and June 2018. A waiver of consent for this analysis was obtained by the Conjoint Faculties Research Ethics Board at the University of Calgary and Alberta Health Services.

### 2.2 Measures

Sex assigned at birth and age at time of treatment were recorded. All patients received a DSM‐5 ED diagnosis by a program physician. The ACE scale (Felitti et al. 1998) is integrated into the standard family assessment for all clients, with the instrument administered by a clinician during a private session with the youth. This scale consists of ten questions, five of which ask about exposure to child abuse or neglect, and the other five, which ask about various forms of household dysfunction (e.g., parental separation). Clinicians administering the ACE advised clients under age 18 that child maltreatment disclosures would need to be reported to child welfare authorities. Each question is scored dichotomously (0 = No; 1 = Yes), so that each item can be analyzed on its own, or the total score can be summed to create a total ACE score (0–10). The outcome of interest was ED treatment completion as reported in clinical case notes.

### 2.3 Statistical Analysis

Data were analyzed using SPSS version 25 (IBM, Armonk, NY, USA) and STATA (StataCorp LLC, College Station, TX, USA). The level of significance was set at 0.05. Maximum likelihood logistic regression (Ramsey and Schafer 2002) models were used to examine the association between patient characteristics and treatment completion status and results are reported as odds ratios (ORs) and 95% confidence intervals (CIs). First the crude association between age, diagnosis, total ACE score and individual ACE items were examined. Then, two separate multivariable models were run: one with total ACE score and the other with individual ACE items, both adjusted for age at start of treatment and DSM‐5 diagnosis. To avoid model instability, variables with *n* < 5 expected observations in at least one cell were collapsed into a smaller number of categories (or removed from the analysis if the raw data were dichotomous).

## 3 Results

Initially, *n* = 218 patients were included in the sample. Each record was examined individually to understand and categorize the reason for file closure: withdrawn (*n* = 71, 33%), completed treatment (*n* = 57, 26%), mutual decision made between patient and clinician that treatment is not in their best interest (e.g., ED is not primary need, treatment fatigue, or other life factors, *n* = 36, 17%), unable to contact or contact information not current (*n* = 19, 9%), transferred to other services (*n* = 15, 7%), declined services without initiating treatment (*n* = 5, 2%), found services elsewhere (*n* = 4, 2%), or Other (*n* = 8, 4%). To simplify the analysis, reporting and interpretation, we decided to restrict our analysis to records where the outcome was clearly identified as either “completed treatment” or “withdrawn,” which yielded a final *n* = 128 (see Table 1 for participant characteristics). Treatment completion was defined as the patient achieving established treatment goals, including but not limited to weight restoration and cessation of symptoms such as compensatory behaviors. Treatment withdrawal was defined as the patient electing to discontinue treatment prior to meeting these goals.

**TABLE 1:** Participant characteristics.

	Full sample (*N* = 128)	Completed treatment (*n* = 57)	Withdrew from treatment (*n* = 71)
	Frequency (%)	Frequency (%)	Frequency (%)
Age			
Mean (SD)	17.4 (3.5)	16.4 (3.2)	18.3 (3.4)
Median (range)	17 (11–24)	16 (11–23)	18 (11–24)
Sex			
Female	121 (95%)	54 (95%)	67 (94%)
Male	7 (5%)	3 (5%)	4 (6%)
Diagnosis			
Anorexia Nervosa restrictive	41 (32%)	22 (39%)	19 (27%)
Bulimia Nervosa	32 (25%)	16 (28%)	16 (23%)
Atypical Anorexia Nervosa	24 (19%)	12 (21%)	12 (17%)
Others	31 (24%)	7 (12%)	24 (34%)
Total adverse childhood experiences score			
Mean (SD)	2.6 (2.5)	1.9 (1.9)	3.2 (2.7)
Median (range)	2 (0–10)	1 (0–7)	3 (0–10)
Verbal abuse	42 (33%)	13 (23%)	29 (41%)
Physical abuse	27 (21%)	6 (11%)	21 (30%)
Sexual abuse	14 (11%)	4 (7%)	10 (14%)
Emotional neglect	56 (44%)	17 (30%)	39 (55%)
Physical neglect	15 (12%)	3 (5%)	12 (17%)
Parental separation	50 (39%)	20 (35%)	30 (42%)
Witnessing intimate partner violence	17 (13%)	3 (5%)	14 (20%)
Substance abuse in home	37 (29%)	10 (18%)	27 (38%)
Mental illness in home	68 (53%)	31 (54%)	37 (52%)
Household member incarcerated	7 (6%)	2 (4%)	5 (7%)

The univariate analysis showed that ACE score, ED diagnosis and age were significantly associated with treatment completion status (Table 2). Due to small number of observations in some of the diagnostic categories, the less common diagnoses in the dataset (avoidant restrictive food intake disorder, unspecified feeding or ED, and other specified feeding or ED), were grouped together as “Other” for the analysis. Sex assigned at birth was removed from the analysis due to inadequate cell count (too few males, *n* = 7, 5%). When adjusted for other covariates simultaneously, the associations of age, diagnosis and ACE score remained significant. People were less likely to complete treatment if they had a higher ACE score (adjusted OR 0.76; 95% CI 0.63, 0.91; *p* = 0.002) or were older (adjusted OR 0.82; 95% CI 0.72, 0.93; *p* = 0.002). The three most common ED categories in the dataset had significantly higher odds of treatment completion than the other (less common) categories. Treatment completion rates was highest for Atypical‐Anorexia Nervosa (adjusted OR 11.54; 95% CI 1.97, 67.55; *p* = 0.007), followed by Bulimia Nervosa (adjusted OR 11.00; 95% CI 2.02, 59.85; *p* = 0.006).

**TABLE 2:** Logistic regression showing association between age, diagnoses, and adverse childhood experiences with likelihood to complete treatment.

	Crude OR (95% CI)	*p*	Adjusted OR (95% CI)	*p*
Age at start of treatment	0.85 (0.76, 0.95)	0.003**	0.82 (0.72, 0.93)	0.002**
Diagnosis				
Anorexia Nervosa	3.67 (0.86, 15.67)	0.120	3.95 (0.87, 18.05)	0.076
Bulimia Nervosa	2.98 (0.75, 11.81)	0.080	11.00 (2.02, 59.85)	0.006**
Atypical Anorexia Nervosa	3.67 (0.81, 16.54)	0.091	11.54 (1.97, 67.55)	0.007**
Others	Reference	Reference	Reference	Reference
Total adverse childhood experiences score	0.80 (0.68, 0.94)	0.006**	0.76 (0.63, 0.91)	0.002**

Abbreviations: CI, confidence interval; OR, odds ratio.

** *p* < 0.01.

Table 3 presents the associations between nine specific ACE items and treatment completion. The ACE item for household member incarceration was excluded from the analysis because of too few endorsements (*n* = 7, 5%). The crude associations of verbal abuse, physical abuse, emotional neglect, witnessing intimate partner violence, and substance abuse in home with treatment completion were statistically significant (*p* < 0.05), with OR ranging from 0.22 to 0.43, meaning that patients exposed to these ACE items were less likely to complete treatment. However, after adjusting for age, diagnosis, and each of the other ACE items, we found that the associations were no longer statistically significant (*p* ≥ 0.056).

**TABLE 3:** Logistic regression showing association between individual items on the adverse childhood experiences scale and likelihood to complete treatment.

	Crude OR (95% CI)	*p*	Adjusted OR (95% CI)	*p*
Verbal abuse	0.43 (0.20, 0.93)	0.033*	1.53 (0.35, 6.74)	0.575
Physical abuse	0.28 (0.10, 0.75)	0.012*	0.47 (0.08, 2.88)	0.415
Sexual abuse	0.45 (0.13, 1.53)	0.202	1.77 (0.35, 8.92)	0.492
Emotional neglect	0.35 (0.17, 0.73)	0.005**	0.32 (0.10, 1.05)	0.061
Physical neglect	0.27 (0.07, 1.00)	0.051	0.51 (0.09, 3.00)	0.458
Parental separation	0.74 (0.36, 1.52)	0.409	1.60 (0.54, 4.73)	0.392
Witnessing intimate partner violence	0.22 (0.06, 0.82)	0.024*	0.18 (0.03, 1.05)	0.056
Substance abuse in home	0.35 (0.15, 0.80)	0.013*	0.82 (0.27, 2.54)	0.733
Mental illness in home	1.10 (0.54, 2.20)	0.798	1.45 (0.59, 3.56)	0.414

Abbreviations: CI, confidence interval; OR, odds ratio.

** *p* < 0.01.

* *p* < 0.05.

## 4 Discussion

To our knowledge, this is the first study to examine how a wide range of child adversities are associated with likelihood to complete ED treatment. Overall, the results support our hypothesis that participants exposed to more types of childhood adversity (those with higher ACEs) were less likely to complete treatment (Table 2), although no individual ACE emerged as an independent predictor of treatment completion after adjusting for diagnosis, age, and exposure to other ACEs (Table 3).

While there is sizable research examining childhood adversity in the etiology of EDs, the body of literature examining treatment retention is limited (Convertino and Mendoza 2023; Day et al. 2024). A key strength of this research is that it includes adolescents and young adults (age range = 11–24), whereas many previous studies of treatment completion only include adults (Convertino and Mendoza 2023; Day et al. 2024). In a longitudinal study assessing the relationships between child maltreatment (physical, sexual, and emotional abuse and neglect) and long‐term treatment outcomes in adults with Anorexia Nervosa and Bulimia Nervosa, patients endorsing a history of child abuse were more likely to drop out of treatment and require hospitalization (Castellini et al. 2018). Likewise, Mahon, Bradley, et al. (2001) found that adult women with Bulimia Nervosa were more likely to drop out of treatment if they experienced sexual abuse or parental separation/divorce (compared to those who did not experience such events). Additionally, Rodríguez et al. (2005) found that adolescent and adult women diagnosed with Anorexia Nervosa, Bulimia Nervosa or Binge Eating Disorder were less likely to complete ED treatment if they were exposed to sexual or physical violence as a child, and that multiple exposures further increased this risk (up to 10‐fold increase in likelihood to drop out). However, other studies in adults have failed to find an association between child abuse and treatment drop out (Carter et al. 2006).

This is the first study to show that total ACE score is negatively associated with ED treatment completion in adolescents. A recent systematic review on adolescent treatment adherence (for any condition) identified three studies on the impact of ACEs, with two studies showing that higher ACE scores were associated with reduced treatment adherence, and one study finding no association (Draxler and Ruppar 2022). In the present dataset, participants were less likely to complete treatment if they were exposed to one of five ACE items: verbal abuse, physical abuse, emotional neglect, witnessing intimate partner violence, and substance abuse in the home (Table 3). However, when we controlled for the effects of age, ED diagnosis, and responses to other ACE questions, there was no significant association between any individual ACE item and likelihood to complete treatment. Considering that four of these five ACE items are considered child maltreatment, while the fifth (substance abuse in home) is a strong predictor of child maltreatment (Doidge et al. 2017), our results could be interpreted as suggesting that child maltreatment is associated with treatment adherence in our ED program. At the same time, since individual categories of childhood maltreatment are correlated (Felitti et al. 1998), it is challenging to identify the unique contribution of different types of child maltreatment toward a given outcome, although this is sometimes possible: for example, Cecil et al. (2017) found that five types of abuse and neglect were individually associated with symptoms of mental illness among high‐risk youth, but emotional abuse showed the strongest association with symptoms when controlling for other types of abuse and neglect. Our data add to an existing literature that suggest researchers should examine a broad spectrum of potentially traumatic events when studying the relationship between childhood adversity and treatment outcomes to improve specificity and reduce the likelihood of spurious correlations.

## 5 Limitations

Younger clients reported fewer ACEs, which may reflect a hesitance to disclose maltreatment exposure due to mandatory reporting laws, or may reflect the fact that older clients have had more time to be exposed to ACEs. Although a cumulative impact of ACEs was observed, this study may have been underpowered to detect the impact of individual ACE items. Additionally, it is unclear whether patients with higher ACE scores were more likely to drop out of treatment because their trauma exposure altered the way they respond to treatment or because of a third variable that is associated with both higher ACE scores and treatment drop out (e.g., financial instability, housing instability, or transportation challenges). Our finding that people with Bulimia Nervosa and Atypical Anorexia Nervosa had higher treatment completion rates is limited by the fact that we were forced to aggregate diagnoses with distinct symptom profiles. Likewise, the fact that treatment plans were individualized for each client meant that were we unable to assess whether treatment modality impacted treatment outcome. The small number of males in our sample (*n* = 7) meant that we were unable to examine the relationship between sex and treatment completion. Previous research highlights that childhood adversity, particularly sexual abuse and physical neglect, contributes to an increased likelihood of developing an ED for males (Afifi et al. 2017). We did not assess for the presence of psychiatric comorbidities in our sample, which may be important since the association between childhood adversity and EDs may be mediated by psychiatric comorbidities (Guillaume et al. 2016).

## 6 Conclusion

The results of this study suggest that adolescents and young adults with EDs are less likely to complete specialized ED treatment if they have exposure to multiple types of childhood adversity. Clinicians providing ED services may measure ACE score before initiating treatment, as is recommended by recent international consensus (Austin et al. 2023). Early intervention in EDs improves outcomes, and supporting youth‐friendly care has been identified as a key component of early intervention in EDs (Allen et al. 2023). The fact that younger patients in our study were more likely to complete treatment than older patients further underscores the importance of initiating ED treatment as soon as possible. As one part of an early intervention strategy for EDs, clinicians could utilize trauma‐informed strategies to improve engagement for youth, such as implementing principles of trauma‐informed care as outlined by the Substance Use and Mental Health Services Administration (2014), including prioritizing physical and emotional safety, building trust with clients through transparent procedures, and involving youth and families in designing their treatment plan.

## Author Contributions

**David Lindenbach:** data curation, formal analysis, investigation, methodology, project administration, supervision, visualization, writing – original draft, writing – review and editing. **Amelia Austin:** writing – original draft, writing – review and editing. **Manya Singh:** formal analysis, investigation, methodology, project administration, writing – original draft, writing – review and editing. **Abigail Trebilcock:** conceptualization, data curation, formal analysis, funding acquisition, investigation, methodology, project administration, writing – review and editing. **Asmita Bhattarai:** formal analysis, investigation, methodology, visualization, writing – original draft, writing – review and editing. **Gisele Marcoux:** conceptualization, data curation, funding acquisition, investigation, methodology, resources, supervision, writing – review and editing. **Gina Dimitropoulos:** conceptualization, funding acquisition, investigation, methodology, resources, supervision, writing – original draft, writing – review and editing.

## Ethics Statement

A waiver of consent for this analysis was obtained by the Conjoint Faculties Research Ethics Board at the University of Calgary and by Alberta Health Services.

## Conflicts of Interest

The authors declare no conflicts of interest.

## Data Availability

The data that support the findings of this study are available on request from the corresponding author. The data are not publicly available due to privacy or ethical restrictions.
